# Update on intravitreal anti-tumor necrosis factor alpha therapies for ocular disorders

**DOI:** 10.1186/s12348-014-0026-8

**Published:** 2014-10-15

**Authors:** Isabel Pascual-Camps, Pablo Hernández-Martínez, Laura Monje-Fernández, Rosa Dolz-Marco, Roberto Gallego-Pinazo, Lihteh Wu, J Fernando Arévalo, Manuel Díaz-Llopis

**Affiliations:** 1 Department of Ophthalmology, University and Polytechnic Hospital La Fe, Av. Fernando Abril Martorell, n° 106, Valencia, 46026, Spain; 2 Department of Ophthalmology, University Hospital Complex, Alto de Nava s/n, Leon, 24071, Spain; 3 Instituto de Cirugía Ocular, Paseo Colón, San José, PO BOX 3971-1000, Costa Rica; 4 Retina Division, Wilmer Eye Institute, Johns Hopkins University School of Medicine, 600 N Wolfe St, Baltimore 21287, MD, USA; 5 Vitreoretinal Division, King Khaled Eye Specialist Hospital, Al Arubah Rd, Umm AL Hamam AL Gharbi, Riyadh 12329, Saudi Arabia; 6 Faculty of Medicine, University of Valencia, Av Blasco Ibañez, 15, Valencia, 46010, Spain

**Keywords:** Tumor necrosis factor alpha, Intravitreal injection, Ocular disorders, Adalimumab, Etanercept, Infliximab

## Abstract

Tumor necrosis factor alpha (TNF-?) is an important pro-inflammatory cytokine associated with a variety of ocular diseases. The currently available TNF-? inhibitors are etanercept, infliximab, adalimumab, golimumab, and certolizumab. Experimental and clinical studies on the intravitreal use of these agents have been reported with etanercept, infliximab, and adalimumab: etanercept has shown limited efficacy in scarce reports; infliximab has been associated with local safety concerns but appears to benefit certain cases; adalimumab has shown no efficacy in cases of age-related macular degeneration (AMD) or diabetic macular edema (DME), but the combination with bevacizumab may be effective in refractory cases of macular diseases. Further preclinical and clinical studies are warranted in order to be able to obtain a more robust conclusion on the use of intravitreal TNF-? inhibitors.

## Review

### Introduction

Inflammation has been implicated as one of the pivotal mediators of retinal injury in the pathogenesis of most sight-threatening ocular diseases including uveitis, diabetic retinopathy and diabetic macular edema (DME), and atrophic and neovascular age-related macular degeneration (AMD) [1],[2]. Thus, a variety of anti-inflammatory and immunomodulatory agents have been tested as potential single or combination therapies in the management of intraocular inflammation, hyperpermeability, and neovascularization. Traditionally, corticosteroids have been the standard approach to this inflammatory pathway inhibition. However, their systemic and ocular safety profiles limit their use [3].

Tumor necrosis factor alpha (TNF-?) is an important pro-inflammatory cytokine with pleiotropic functions synthesized mainly by T lymphocytes and macrophages and to a lesser extent by neutrophils and mast cells [4]. It plays a major role in the regulation of immune cells, inhibition of tumorigenesis, and inhibition of viral replication [5]-[8].

The currently available TNF-? inhibitors are etanercept, infliximab, adalimumab, golimumab, and certolizumab. Etanercept (Enbrel; Pfizer Inc; New York, NY, USA) is a TNF receptor-IgG fusion protein that mimics the inhibitory effects of naturally occurring soluble TNF receptors that is injected subcutaneously. Infliximab (Remicade; Schering-Plough, Rathdrum, Ireland) is a mouse-human chimeric antibody that neutralizes the biological activity of TNF-? by high-affinity binding to the soluble and transmembrane forms of TNF-?, therefore preventing the effective binding of TNF-? with its receptors. It is administered intravenously. Adalimumab (Humira; AbbVie Inc., North Chicago, IL, USA) is a fully human monoclonal antibody that also binds selectively all forms of TNF-?. It is also injected subcutaneously. Golimumab (Simponi; Centocor, Horsham, PA, USA, and Schering-Plough, Rathdrum, Ireland) is a fully human monoclonal antibody against TNF-? that is administered via a subcutaneous injection. Certolizumab (Cimzia; UCB Pharma, Brussels, Belgium) is a monoclonal antibody that combines the Fab fragment of the TNF antibody with polyethylene glycol that is delivered subcutaneously. Table 1 summarizes the main features of these molecules.

**Table 1:** Summary of the main features of the currently available tumor necrosis factor alpha inhibitors

**Generic name (brand name)**	**Molecular weight (kDa)**	**Mechanism of action**	**Route of administration**	**Half-life (days)**
Etanercept (Enbrel)	150	TNF soluble decoy receptor	Subcutaneous injection	4 to 6
Infliximab (Remicade)	149	Anti-TNF monoclonal antibody	Intravenous infusion	8 to 10
Adalimumab (Humira)	148	Anti-TNF monoclonal antibody	Subcutaneous injection	14
Golimumab (Simponi)	150 to 151	Anti-TNF monoclonal antibody	Subcutaneous infusion	14
Certolizumab (Cimzia)	91	Pegylated anti-TNF monoclonal antibody	Subcutaneous injection	14

kDa, kilodalton; TNF, tumor necrosis factor.

Reported risks of the systemic administration (intravenous or subcutaneous) of TNF-? inhibitors include fatal blood disorders, secondary infections, and reactivation of latent infections, tumorigenesis, drug-induced lupus, or demyelinating central nervous system disorders among others. These potentially severe adverse events led to the investigation by ophthalmologists of alternative administration routes minimizing these risks but preserving the efficacy of the drugs. The possibility of performing intravitreal injection of TNF-? inhibitors could fulfill both needs [9]. However, no well-designated trials have been conducted to date [9]-[11], and the use of the intravitreal route of administration of TNF-? inhibitors has not been generalized.

The purposes of the present review are to analyze the current published evidence with regard to intravitreal injection of TNF-? inhibitors and to summarize the outcomes with this novel therapeutic approach.

### Methods

A systematic review of all the peer-reviewed articles indexed in PubMed was performed. A comprehensive search of the literature was conducted using the online biomedical search engine PubMed. Search terms included the following: intravitreal, etanercept, infliximab, adalimumab, golimumab, certolizumab, tumor necrosis factor inhibitors. No publication date limit was applied, thus including all the available reports. Preclinical experimental models, clinical case reports, pilot studies, and case series were reviewed independently for the intravitreal use of etanercept, infliximab, and adalimumab. Relevant articles cited in papers retrieved from PubMed were also reviewed. No preclinical or clinical experience with intraocular administration of golimumab or certolizumab was found.

#### Intravitreal experience with etanercept

Experimental animal models showed that intravitreal injections of etanercept up to 2.5 mg may be well tolerated without significant toxic effects on the retina [12],[13].

Clinical experience has been limited to a small pilot study that included seven patients with refractory DME [14]. Following two intravitreal injections of 2.5 mg (0 and 1 mL) of etanercept with a 2-week interval, no adverse event was registered, obtaining no significant improvement in 3 months after initiation of this treatment [14]. There is no scientific background that may reinforce the use of intravitreal injections of etanercept for the management of ocular diseases.

#### Intravitreal experience with infliximab

Experimental animal models showed that intravitreal injections of infliximab were tolerated up to 2.0 mg. However, doses higher than 3.3 mg may be potentially toxic to the retina [15]-[20]. Also, Regatieri et al. showed in a laser-induced choroidal neovascularization model that intravitreal infliximab when injected at doses of 10 to 40 ?g has an anti-angiogenic effect, whereas higher doses (320 ?g) showed pro-angiogenic effects [21]. More recently, Yuksel et al. evidenced in a rabbit model of experimental endotoxin-induced uveitis that intravenous administration of infliximab was more effective than the intravitreal route in an acute period [22].

Beer et al. suggested that infliximab may be suitable for compounding and could be a cost-effective intravitreal medication for use in clinical practice [23]. A summary of the clinical experience reported is provided in Table 2.

**Table 2:** Summary of publications regarding the use of intravitreal infliximab

**Publications**	**Indication**	**Dosing**	**Number of cases**	**Main results**
Theodossiadis et al. [17]	Neovascular AMD	2.0 mg/0.05 mL	3	BCVA improved from 20/200 to 20/40 after two monthly injections in case 1, from 20/200 to 20/70 after two bimonthly injections in case 2, and from 20/100 to 20/30 after two injections with a 3-month interval.
Giganti et al. [25]	Neovascular AMD	0.5 mg/0.05 mL	2	At week 12, BCVA had declined in three patients, and electroretinography and microperimetry had a decrease in all cases.
	DME	0.5 mg/0.05 mL	2	
Arias et al. [26]	Neovascular AMD	2.0 mg/0.05 mL	4	At 3 months after a single injection, the BCVA change was ?18, +3, +4, and ?4 letters.
				Two cases developed severe intraocular inflammation.
Wu et al. [27]	DME	1.0 mg/0.05 mL	15	BCVA changed from 1.49 ± 0.58 to 1.38 ± 0.56 logMAR at 3 months in the 1 mg group.
		2.0 mg/0.10 mL	19	BCVA changed from 0.76 ± 0.5 to 1.03 ± 0.7 logMAR at 3 months in the 2 mg group, and 42% of cases developed severe intraocular inflammation.
Wu et al. [28]	Neovascular AMD	1.0 mg/0.05 mL	8	BCVA changed from 1.04 ± 0.2 to 1.06 ± 0.5 logMAR at 3 months in the 1 mg group.
		2.0 mg/0.10 mL	8	BCVA changed from 0.94 ± 0.5 at baseline to 0.85 ± 0.4 logMAR in the 2 mg infliximab group.
				37.5% of eyes developed severe intraocular inflammation.
Farvardin et al. [29],[30]	NIPU	1.5 mg/0.15 mL	7	BCVA changed from 1.37 ± 0.4 to 1.38 ± 0.4 logMAR at 6 months.
				Vitreous haziness changed from 2.7 to 2.6 at 6 months.
Markomichelakis et al. [31]	Behçet	1.0 mg/0.05 mL	15	BCVA changed from 0.74 to 0.3 logMAR at 30 days.
				Intraocular inflammation decreased through day 30.
Wu et al. [32]	PCME	1.0 mg/0.10 mL	7	BCVA changed from 1.14 ± 0.6 to 0.51 ± 0.35 logMAR.
				Mild intraocular inflammation was observed in one case.

AMD, age-related macular degeneration; DME, diabetic macular edema; NIPU, noninfectious posterior uveitis; PCME, pseudoaphakic cystoid macular edema; BCVA, best-corrected visual acuity; logMAR, logarithm of the minimal angle of resolution.

Theodossiadis and colleagues treated three patients with exudative AMD unresponsive to ranibizumab with two injections of 2 mg of infliximab given 2 months apart [24]. They reported visual improvement in all three cases (20/200 to 20/40; 20/200 to 20/70; and 20/100 to 20/30) with no safety concerns. In contrast, Giganti et al. reported that intravitreal infliximab at a dose of 0.5 mg in two eyes of DME and in two cases of neovascular AMD was associated with anatomic, electroretinographic, and microperimetric worsening, and three of these cases developed intraocular inflammation [25]. Arias et al. conducted a prospective interventional trial of four eyes with exudative AMD refractory to anti-vascular endothelial growth factor (VEGF) agents. They reported that intravitreal infliximab at a dose of 2 mg did not provide any visual or anatomic benefit. Furthermore, half of their patients developed a severe intraocular inflammatory reaction [26]. The Pan-American Collaborative Retina Study Group has published the largest series on the use of intravitreal infliximab in both DME and exudative AMD. In their series, eyes with refractory DME received a single intravitreal injection of infliximab 1.0 mg/0.05 mL (15 eyes) or 2.0 mg/0.10 mL (19 eyes). No significant anatomical or functional benefit was evidenced. In addition, 42% of eyes injected with 2 mg developed a severe uveitis, and three of these eyes (37.5%) required pars plana vitrectomy [27]. Similar results were found in 26 eyes with partially responsive neovascular AMD to intravitreal injections of VEGF inhibitors. No functional gains were seen, and in addition, 37.5% of patients developed a severe uveitis that resolved with intensive topical steroid therapy [28].

Farvardin et al. reported positive both short- and long-term (6 months) outcomes after intravitreal infliximab 1.5 mg/0.15 mL in ten eyes of seven patients with chronic persistent noninfectious uveitis without any ocular adverse event, showing a visual mean visual improvement from 1.37 logMAR to 0.67 logMAR 1 month after the injection, which was not sustained by month 6 due to recurrence of macular edema and vitreous haze [29],[30].

Markomichelakis et al. reported that a single intravitreal injection of infliximab 1.0 mg/0.05 mL controlled intraocular inflammation in 15 cases with relapsing posterior uveitis associated with Behçet's disease [31].

Wu et al. found that a single intravitreal injection of infliximab 1.0 mg/0.10 mL achieved excellent outcomes after 6 months, without any intraocular inflammation, in seven cases of refractory pseudophakic cystoid macular edema [32].

#### Intravitreal experience with adalimumab

Experimental animal models have shown that intravitreal injections of adalimumab were tolerated up to 5.0 mg without electroretinographic or histological alterations [33]. However, doses of 10.0 mg have been associated with early retinal toxicity and a significant decrease in the photopic wave in the electroretinographic response [34],[35].

The Pan-American Collaborative Retina Study Group published their clinical experience with intravitreal injections of adalimumab for patients with refractory DME and partially responsive neovascular AMD to VEGF inhibitors. Intravitreal injections of adalimumab 2.0 mg/0.08 mL did not provide any anatomical or functional benefit in five cases of DME nonresponsive to conventional therapies and four cases of neovascular AMD. No ocular side effects were reported by the authors following intravitreal injections of adalimumab [26],[27].

The possible role of intravitreal adalimumab in combination with anti-angiogenic agents (bevacizumab) has also been analyzed by these authors. In their case series of seven eyes of five patients with macular edema of various etiologies including partially responsive neovascular AMD, a clinically relevant response was achieved after combination intravitreal treatment with adalimumab 2 mg/0.08 mL and bevacizumab 1.25 mg/0.05 mL [36].

## Conclusions

Overall, the literature review of all published manuscripts regarding the use of intravitreal TNF-? inhibitors reveals the following conclusions: 1) intravitreal injections of etanercept may not be useful in the management of ocular diseases, 2) intravitreal injections of infliximab may potentially be related to severe intraocular inflammation, 3) intravitreal injections of infliximab do not appear to benefit cases of refractory DME or partially responsive neovascular AMD, 4) intravitreal injections of infliximab may benefit cases of persistent noninfectious posterior uveitis and refractory pseudophakic cystoid macular edema, 5) intravitreal injections of adalimumab do not appear to benefit cases of DME or neovascular AMD, 6) intravitreal combination of adalimumab and bevacizumab may be effective in the management of cases with partially responsive neovascular AMD and macular edema of various etiologies, and 7) no preclinical or clinical experience with intravitreal golimumab or certolizumab has yet been published.

It is important to emphasize that all the reviewed reports include small samples, variable endpoints and follow-up periods, and heterogeneity in the doses administered, the selection criteria, and the patient population [9]-[11]. The off-label intravitreal use of any drug should be cautiously considered given the potentially severe adverse events that this procedure may induce. Further preclinical and clinical studies are warranted in order to be able to obtain a more robust conclusion on the use of intravitreal TNF-? inhibitors.

## Abbreviations

AMD: age-related macular degeneration

DME: diabetic macular edema

TNF: tumor necrosis factor

VEGF: vascular endothelial growth factor

## Competing interests

The authors declare that they have no competing interests.

## Authors' contributions

All authors have made substantial contributions to the conception and design, acquisition of data, or analysis and interpretation of data; have been involved in drafting the manuscript or revising it critically for important intellectual content; have given final approval of the version to be published; and agreed to be accountable for all aspects of the work in ensuring that questions related to the accuracy or integrity of any part of the work are appropriately investigated and resolved.
